# Evaluating machine learning models for clothing size prediction using anthropometric measurements from 3D body scanning

**DOI:** 10.1038/s41598-025-24584-6

**Published:** 2025-11-19

**Authors:** Ruqey Alhassawi, Simeon Gill, Steven Hayes, Kristina Brubacher

**Affiliations:** Department of Materials, The University of Manchester, Manchester, UK

**Keywords:** 3D body scan, Machine learning, Body measurements, Clothing size prediction, Standard dress form, Engineering, Materials science, Mathematics and computing

## Abstract

An analysis of a dataset comprising 677 participants revealed substantial discrepancies in size categorization. Only 63 individuals (9.15%) maintained consistency across bust, waist, and hip measurements, whereas 614 participants (90.84%) exhibited size variations, and 35.45% were not adequately accommodated by the existing sizing scheme. These findings highlight significant challenges in garment selection, potentially leading to dissatisfaction and increased return rates. This study evaluated the effectiveness of support vector machine (SVM) and principal component analysis-SVM (PCA-SVM) models for clothing size prediction via 3D body scanning data. The traditional SVM model, which focuses on primary measurements, achieves an accuracy of 89.66%, outperforming the PCA-SVM model (68.97%), which incorporates additional dimensions. These results underscore the effectiveness of SVMs in predicting clothing size categories and emphasise the intricate relationship between body morphology and garment fit.

## Introduction

Predicting garment sizes accurately is essential for improving consumer retention, reducing returns, and streamlining inventory control^1^. Traditionally, size prediction has relied on standard sizing charts and a limited range of body measurements, often failing to account for the diversity in human body shapes and proportions^2^.

To address these limitations, the use of 3D body scanning technology offers substantial advantages for the garment industry by facilitating the extraction of accurate body measurements, leading to better-fitting garments through customization and optimised garment development^3^. Researchers have applied 3D body scan technology data to create effective development practices for clothing fitting and classify body forms through the application of advanced algorithms^4,5^.

Machine learning (ML) techniques have emerged as powerful tools for enhancing the accuracy and reliability of size predictions in numerous studies^6–9^. Artificial intelligence (AI) and machine learning are transforming size prediction in the fashion industry by improving accuracy and personalization^10^. These technologies analyse body measurements to offer customised garment size recommendations, surpassing the limitations of traditional sizing methods^11^. Technologies such as support vector machines (SVMs), clustering, neural networks (NNs), regression, and principal component analysis (PCA) enable the integration of multiple measurements, resulting in more accurate size predictions^10,12^. AI and ML play essential roles in advancing fashion technology, making the industry more adaptive and efficient. While this study utilises established machine learning algorithms, its primary contribution lies in applying these methods to empirically examine the challenges of standard sizing. It moves beyond theoretical discussion by demonstrating the effectiveness of analytical algorithms in achieving accurate size prediction.

Support vector machines (SVMs) have gained prominence in this area because of their robustness and efficiency in handling high-dimensional data^6,13^. Initially, introduced by^14^, SVM has emerged as a powerful machine learning technique for classification and regression tasks, including applications in size prediction for the fashion industry^7,12^. Furthermore, a study by Zhang^15^ compared SVM and neural networks for body type identification and concluded that SVM achieved higher accuracy and positively impacted garment production. Zhang’s study highlights SVM’s effectiveness in handling high-dimensional data, demonstrating its ability to predict clothing sizes on the basis of multiple body measurements^15^. Additionally, PCA is widely used in dimensionality reduction, allowing for effective clustering and classification to segment populations into distinct body types^16^. For example, PCA has been applied in various anthropometric studies, including children’s clothing sizing^17^ and female body protector design^18^.

## Literature review

### Traditional sizing systems and dress forms

The standard body dress form has historically served as a foundational tool in clothing product development, providing a reference model for creating garments that accommodate various body types^19,20^. These dress forms are typically developed using average body measurements from population studies to maintain uniformity in mass-produced clothing^21^. The development of apparel sizing systems relies on anthropometric data to generate standard body measurement charts that serve as the foundation for garment size classification^20^. The ISO 8559 series provides standardised methods for defining body measurements and size profiles^22^. Landmark studies, including analysis of 10,213 measurements of American women, played pivotal roles in establishing size series that significantly influenced clothing fit globally^23,24^. However, these traditional methods often fail to account for individual differences in body shape and size, resulting in inconsistencies in garment fit and discrepancies between clothing size labels and actual fit^25^. The diversity of body shapes and proportions cannot be fully captured by standard forms based on simplified average data, leading to ongoing challenges in the garment industry.

### Body measurements and 3D scanning technology

Statistical analysis of body measurement surveys is essential in the formulation of sizing systems, as it considers variables such as control measures, size ranges, body proportions, and size intervals^26^. For women’s ready-to-wear clothing, key measurements including bust circumference for upper-body garments and waist and hip circumferences for lower-body clothing have been identified through variance and factor analysis^26,27^. Another study proposed that bust, waist, abdominal extension, and hip girths serve as effective alternative measurements for garment sizing, with busts being preferred over chest girths and hips being favoured over sitting-spread girths^28^.

The use of 3D body scanning technology has proven effective in addressing garment fit challenges and enhancing clothing design and retail practices^3,29,30^. This technology facilitates mass customization by enabling personalised clothing modifications on the basis of individualised body scans^31^. Additionally, 3D scanning allows for a contactless fit assessment by analysing the space between the garment and the body^32^. With continuous technological advancements, there is a growing need for industry practitioners to adopt data-driven methods in product development. This includes incorporating engineering principles and re-evaluating conventional pattern-making techniques to optimise garment fit and manufacturing efficiency^3^.

### Comparative analysis of machine learning methods in anthropometric classification

Choosing suitable machine learning algorithms for classifying anthropometric data involves evaluating the strengths and limitations of each method. For example, Random Forest (RF) algorithms are commonly applied to model complex non-linear patterns and to evaluate the relative importance of input features^33^. Random Forest algorithms are effective tools for both classification and regression tasks; however, they can encounter challenges when dealing with high-dimensional datasets^34^. Their performance may decline due to the inclusion of irrelevant features and a tendency to favour features with multiple distinct values^35^. Furthermore, Artificial neural networks (ANNs) have gained increasing attention in fashion technology and body measurement applications for their ability to model complex, non-linear relationships^36^. They have shown promising results in predicting garment sizes, ease preferences, and body dimensions using diverse input data such as 3D body scans, key measurement points, and estimated anthropometric features^37,38^. However, the efficiency of (ANNs) in anthropometric implementations is often constrained by the limited size of available datasets, which can lead to overfitting particularly in studies involving small, specialised populations^39,40^. A primary limitation of ANNs is their black box nature referring to the difficulty in understanding or interpreting how the model processes inputs and arrives at its predictions making it challenging to decide which specific body measurements influence the final size classification^41,42^. This lack of transparency reduces their practical applicability in garment design, where explain ability is crucial^43^. Moreover, ANNs require greater computational resources compared to more efficient methods like Support Vector Machines (SVMs), and further evaluation across diverse garment styles and demographic groups is needed to enhance their practical applicability^44^. Studies have shown that SVMs perform well in body type classification, particularly for women, often outperforming neural networks in accuracy and efficiency^15,45^. SVMs also require less training data than tree-based models, making them a practical and interpretable option for garment design^46,47^.

The effectiveness of SVM for anthropometric classification can be attributed to its regularization features that address overfitting challenges in small datasets, flexible kernel transformations that accurately represent non-linear patterns between anthropometric variables, superior computational speed compared to neural network approaches, and documented reliability in apparel size prediction research^12,45,47^. This study employed SVM due to its demonstrated reliability in classification tasks, particularly its capacity to handle non-linear relationships while maintaining computational efficiency and overfitting resistance. SVM’s interpretable support vector framework provides clear insights into decision boundaries, making it well-suited for practical garment sizing applications.

### Support vector machine (SVM) for anthropometric analysis

Support vector machines (SVMs) are versatile machine learning algorithms widely applied to garment size prediction based on human body measurements^6,7,48,49^. SVM classification identifies optimal hyperplanes that maximize the margin between size categories in feature space, where support vectors represent the closest body measurements to decision boundaries^50,51^. In apparel size classification, these hyperplanes serve as decision boundaries, segmenting the high-dimensional anthropometric feature space into distinct size categories.

SVM effectiveness depends on kernel function selection, which defines feature space transformation and decision boundary characteristics. The linear kernel^52^ establishes hyperplane separation via Eq. (1):

1 $${\text{w}} \cdot {\text{x}} + {\text{b}} = 0$$

This kernel is effective for linearly separable data^53^. However, nonlinear classification scenarios employ the kernel trick to map data into higher-dimensional space, enabling complex decision boundaries^51,54^. The radial basis function (RBF) kernel, widely used in nonlinear applications^52^, maps features into infinite-dimensional space, providing flexible decision boundaries mathematically expressed as Eq. (2)

2 $${\text{\rm K}}\left( {{\text{x}},{\text{y}}} \right) = {\text{exp}}\left( { - {\upgamma }\left\| {{\text{x}} - {\text{y}}} \right\|^{2} } \right)$$

While polynomial and sigmoid kernels suit different datasets and classification tasks^54^, this study employed the RBF kernel for its robust performance in handling nonlinear anthropometric data classifications.

### Principal component analysis for anthropometric analysis

In garment sizing applications, body measurement datasets present several challenges. While anthropometric data extracted from 3D body scans can provide comprehensive measurements (including circumferences, heights, widths, and arcs) that capture body diversity more accurately than traditional three-measurement approaches, this high dimensionality can reduce model efficiency and introduce redundant information^55^. Principal component analysis (PCA) addresses this issue by transforming correlated anthropometric variables into principal components that capture maximum variance while reducing dimensionality^56^.

For body measurement analysis, PCA implementation typically involves three stages^17^: (1) standardisation, ensuring uniform contribution across measurements with different scales (e.g., centimetres for height versus circumferences); (2) covariance matrix computation, identifying relationships between measurements (e.g., correlation between bust and shoulder width); and (3) eigenvector and eigenvalue calculation, determining which measurement combinations explain the greatest variation in body shape^56^. This process converts high-dimensional anthropometric data into interpretable components while preserving the variance essential for size classification^16^. When combined with SVM classification, the PCA-SVM approach enables assessment of whether comprehensive body measurement data improves size prediction accuracy compared with traditional key-measurement methods, thereby directly addressing the challenge of accommodating diverse body shapes within standardised sizing systems.

## Methodology

### Data collection

The study utilises data obtained through 3D body scanning technology to collect detailed measurements from 677 female participants. This method captures the entire shape of the body, allowing for measurements of dimensions such as lengths, circumferences, and heights. The data were collected via size stream body scanning technology and software (Cary, NC, USA) and were analysed by the Apparel Design Engineering group at the University of Manchester.

The participants in this study ranged in age from 18 to 77 years, with an average age of 29 years and a median age of 24 years. Their heights ranged from 124.50 cm to 183.60 cm, with an average height of 163.98 cm and a median height of 164.00 cm. Weights varied from 37.10 kg to 163.65 kg, with a mean weight of 61.37 kg and a median weight of 59.50 kg. The ethnic composition of the sample was diverse, including 59% White, 34.9% Asian (including 23.6% Chinese and 11.3% other Asian backgrounds), 1.4% Black, 0.5% Mixed White and Black, 1.4% other mixed backgrounds, 0.9% other ethnic groups, and 0.9% undisclosed ethnicity.

## Research design

**Fig. 1:**
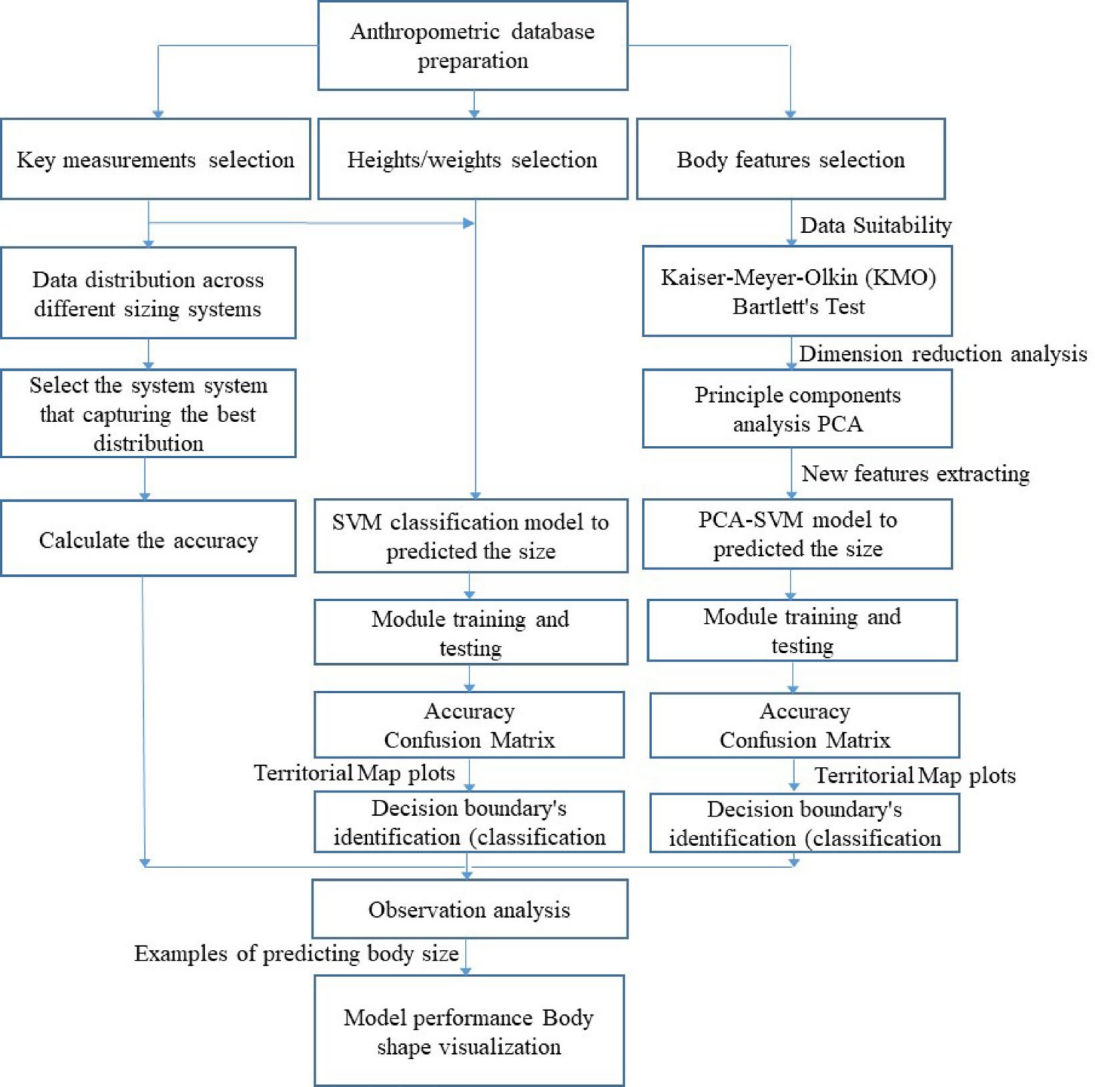
Research framework.

This study employs a quantitative and computational approach to assess the effectiveness of machine learning models in clothing size prediction. It follows a structured process (Fig. 1), beginning with data collection via 3D body scanning, followed by manual size classification via multiple sizing systems. Key body measurements are then selected and analysed to develop and train the SVM and PCA-SVM models. Finally, the models’ accuracy and performance are evaluated through statistical analysis, ensuring a systematic and data-driven assessment of size prediction methods.

### Sizing systems and measurement selection

Three sizing systems were utilised in this analysis: the University of Manchester (UOM) system, modified by the Apparel Design Engineering Group; the UK Alvanon system; and the commercial JD Williams system. These systems were selected on the basis of their distinct characteristics: UOM’s academic foundation, Alvanon’s industry-standard approach, and JD Williams’ consistent size gradation. The comparative analysis evaluated each system’s ability to classify a wide range of body measurements accurately within their respective size intervals.

**Fig. 2:**
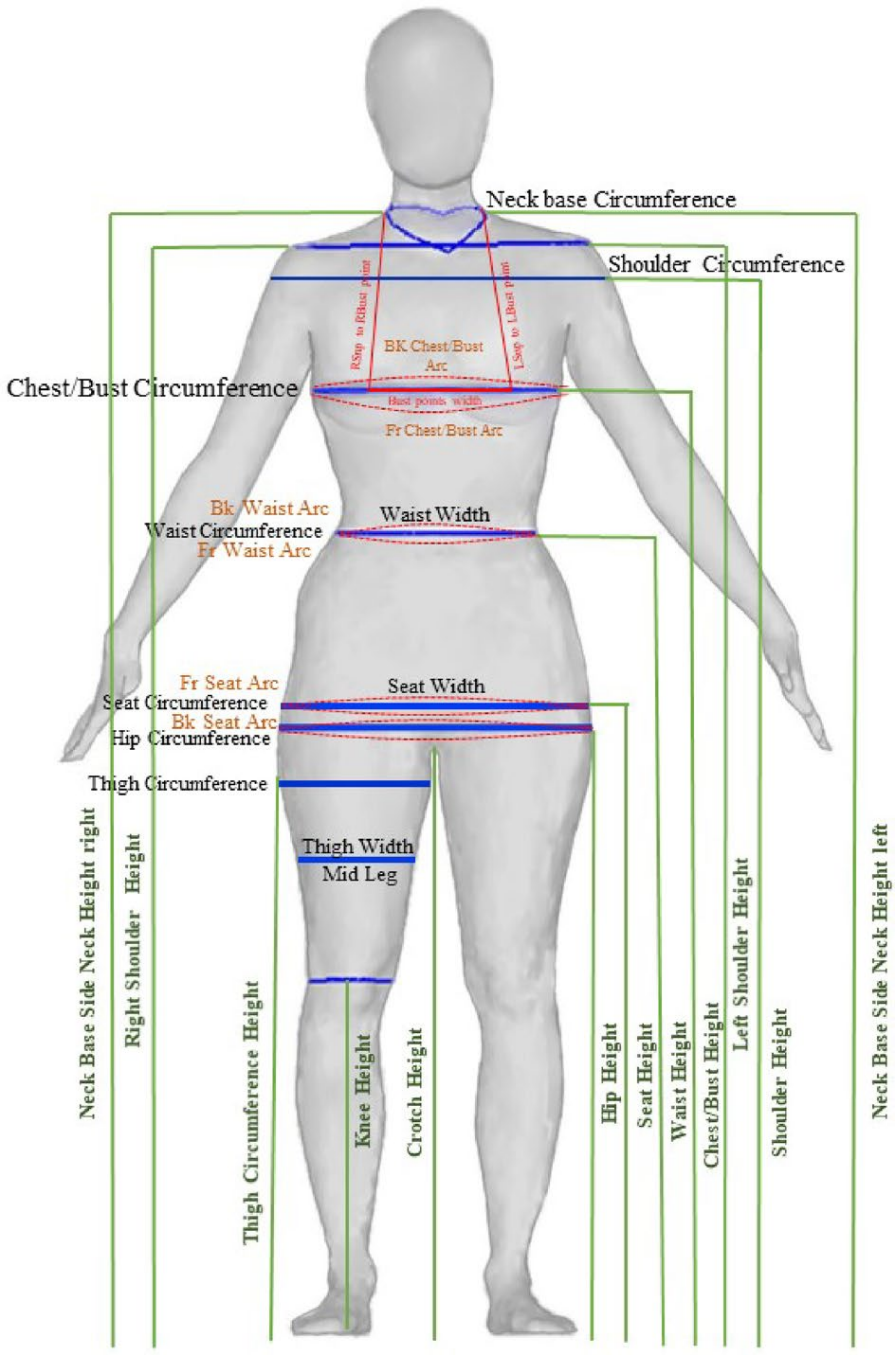
Body measurements.

Figure 2 illustrates key circumferential measurements (chest/bust, waist, hip) along with secondary dimensions (widths, arcs, height segments) that are critical for garment sizing. By integrating both horizontal and vertical measurements, this study examines the effectiveness of using height and weight alone previously identified by^28^ as strong predictors of body dimensions compared with additional measurements for enhanced body size prediction. This visualization supports the study’s SVM-based methodology, which aims to accommodate a diverse range of body shapes within sizing systems.

### Model development for body size production

In body dimension classification for clothing size prediction, the radial basis function (RBF) kernel is often preferred because of its ability to model complex, nonlinear relationships between body measurements and size categories^57^. The RBF kernel is a popular option for nonlinear classification tasks because it projects input features into a higher-dimensional space where linear separation is not feasible^52^. The RBF kernel function is defined by Eq. (2), where x and y represent the input feature vectors, γ (gamma) is a parameter that determines the spread of the kernel and influences the complexity of the decision boundary, and $\Vert \text{x}-{\text{y}\Vert }^{2}$ is the squared Euclidean distance between the samples x and y^51,57^.

The SVM implementation in MATLAB employs the radial basis function (RBF) kernel within an error-correcting output codes (ECOC) framework, enabling multiclass classification of anthropometric measurements into distinct size categories.


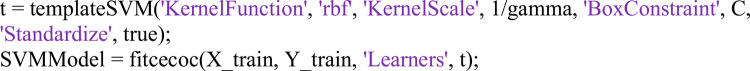


The ‘templateSVM’ function creates an SVM template with an RBF kernel, specified by the ‘KernelFunction’ parameter, for use in multiclass classification with ‘fitcecoc’. The ‘KernelFunction’ parameter determines the kernel type, which in this case is set to “rbf” for the radial basis function. The ‘Standardize’ option centers and scales predictors, enhancing model consistency^58^.

The ‘fitcecoc’ function applies the error-correcting output codes (ECOC) framework, which extends binary SVM to multiclass classification tasks by decomposing them into multiple binary classification problems. The ‘Learners’ parameter defines the SVM template for each binary classifier in the ECOC model. The ECOC approach^59^ encodes multiclass distinctions as binary tasks, producing code words from binary outputs that correspond to predefined class labels, thereby enabling efficient multiclass classification.

### PCA-SVM model

Principal component analysis (PCA) was applied to reduce dataset dimensionality and identify significant features representing body size and shape. Before implementing PCA, dataset suitability was assessed using two statistical tests. The Kaiser–Meyer–Olkin (KMO) measure evaluates sampling adequacy, with values ≥ 0.6 considered acceptable for factor analysis^60^. Bartlett’s test of sphericity assesses whether significant correlations exist among variables, with p < 0.05 confirming that PCA is an appropriate dimensionality reduction technique^61^. The PCA implementation identified principal components accounting for high cumulative variance, indicating that these components retained the majority of information from the original body measurements. This process substantially reduced dataset dimensionality while preserving variability essential for size classification. The rotated component matrix provided insight into the underlying structure of body measurements by displaying the loadings of each original variable on the identified principal components. Component loadings greater than 0.50 were considered significant for factor interpretation, with the highest loading variables selected as representative features for each principal component^60^.

Model validation employed a 70:30 training-to-testing split using MATLAB’s cvpartition function to assess SVM and PCA-SVM generalizability in body size prediction. Model performance was assessed using two metrics. Accuracy quantifies the proportion of correct predictions relative to total test cases (Eq. 3). Confusion matrices provide detailed breakdowns of true and false predictions across all size categories, highlighting misclassifications and enabling quantitative assessment of classification effectiveness for both models.

3 $${\text{Accuracy}} = \frac{{{\text{True}}\;{\text{predictions}}\;{\text{in}}\;{\text{the}}\;{\text{test}}\;{\text{set}}}}{{{\text{total}}\;{\text{predictions}}\;{\text{in}}\;{\text{the}}\;{\text{test}}\;{\text{set}}}} \times 100$$

Decision boundaries were visualized by constructing mesh grids spanning the feature space. Both SVM and PCA-SVM models classified each grid point, generating color-coded scatter plots displaying predicted labels and size centroids. Additionally, 3D body scan data were compared with 2D representations of circumferences and heights, simplifying complex body shapes into outlines. This visualization approach evaluated how effectively both models capture body size variations and their practical applicability in garment sizing systems.

## Results

### Distribution of research participants across different size systems

Table 1 presents the distribution of research participants across various size intervals for three sizing systems: UOM, Alva UK, and JD Williams. Each row represents a specific size range, while the columns display the number of participants falling into that category on the basis of different body measurement combinations: whole body (bust, waist, and hip), upper body (bust and waist), lower body (waist and hip), and torso (bust and hip).

**Table 1:** Interval distribution of research participants by size system interval.

Size system	Size	Whole body Bust, Waist, and Hip	Upper body Bust and Waist	Lower body Waist and Hip	Torso Bust and Hip
Illustration					
UOM size system	6	1	1	1	1
	8	2	6	11	6
	10	21	31	64	39
	12	22	56	68	46
	14	7	29	45	31
	16	2	12	22	13
	18	1	2	6	7
	20	0	2	3	1
	22	0	2	8	2
	24	0	2	0	0
	N	56	143	228	146
Dress form UK Alva size system	6	1	2	1	1
	8	2	8	12	9
	10	22	32	60	41
	12	26	58	79	54
	14	7	26	51	30
	16	0	8	24	11
	18	2	4	6	6
	20	0	2	4	1
	22	0	3	7	3
	24	0	3	0	0
	N	59	145	243	155
JD Williams Size System	6	0	3	3	0
	8	7	30	31	14
	10	15	47	52	38
	12	19	39	63	40
	14	9	14	29	26
	16	0	4	11	5
	18	0	3	6	1
	20	0	1	5	2
	22	2	2	3	4
	24	0	1	0	0
	N	52	144	203	130

The data revealed significant variation in participant numbers across size categories, particularly when different body measurement combinations were considered. Notably, size 12 consistently has the highest participant count across all measurement categories. Among the three systems, the Dress Form UK Alva size system has the largest number of cases across all body shapes, with 139 participants recorded as size 12.

The UK Alvanon system effectively captures the distribution of participants by not only recording the highest number for size 12 but also maintaining a well-balanced distribution across other size categories. On the basis of these findings, the Dress Form UK Alva Size System emerges as the most informative and reliable framework for analysing size distribution in this research.

### Alvanon UK sizing system

This study utilises the UK Alvanon dress form sizing system (Table 2)^62^, which applies a consistent 5 cm grading interval for bust, waist, and hip measurements across all sizes. Each size is defined by a central measurement with a ± 2.5 cm tolerance, creating contiguous size ranges. This standardised approach facilitates the systematic analysis of anthropometric variations in analyses and enhances the accuracy of size prediction models.

**Table 2:** UK Alvanon size category intervals in cm.

Size	Bust range	Waist range	Hip range
6	75 ± 2.5	57 ± 2.5	83 ± 2.5
8	80 ± 2.5	62 ± 2.5	88 ± 2.5
10	85 ± 2.5	67 ± 2.5	93 ± 2.5
12	90 ± 2.5	72 ± 2.5	98 ± 2.5
14	95 ± 2.5	77 ± 2.5	103 ± 2.5
16	100 ± 2.5	82 ± 2.5	108 ± 2.5
18	105 ± 2.5	87 ± 2.5	113 ± 2.5
20	110 ± 2.5	92 ± 2.5	118 ± 2.5
22	115 ± 2.5	97 ± 2.5	123 ± 2.5
24	120 ± 2.5	102 ± 2.5	128 ± 2.5
Grading	5 cm	5 cm	5 cm

The results presented in Table 3 indicate that not all participants fell within the same size interval for all three primary measurements. Only 63 individuals (9.15%) were consistently classified within the same size category for bust, waist, and hip circumferences. In contrast, 614 participants (90.84%) exhibited variations across these key measurements.

**Table 3:** Populations’ ability to match the expectations of the sizing system.

	Example of Garment and required measurements							
Size	N participants			Bust, Waist, and Hip	Bust and Waist	Waist and Hip	Bust and Hip	
Illustration	Bust	Waist	Hip					Total
4	0	0	2	–	–	–	–	–
6	12	4	3	1	2	1	1	2
8	54	33	43	2	8	12	9	25
10	126	114	137	22	32	60	41	89
12	173	181	161	26	58	79	54	139
14	130	142	144	7	26	51	30	93
16	73	78	89	0	8	24	11	43
18	42	38	35	2	4	6	6	12
20	22	28	19	0	2	4	1	7
22	16	20	21	0	3	7	3	13
24	10	10	7	0	3	0	0	3
26	6	9	4	0	1	1	0	2
28	5	7	6	2	2	2	3	3
30	4	5	2	0	1	0	1	2
32	4	4	2	1	2	1	2	3
34	0	4	1	0	0	1	0	1
36	0	0	0	–	–	–	–	–
38	0	0	0	–	–	–	–	–
40	0	0	1	–	–	–	–	–
%s that meet the size interval	677	677	677	63 /9.15% (614/90.84%)	152	249	162	437(64.55%) /240 (35.45% no size)

Furthermore, a substantial proportion of participants (437 individuals, 64.55%) matched at least two of the primary measurement intervals. The total number of participants sharing at least two key measurements varied across sizing categories as follows: 63, 152, 249, and 162 individuals within sizes 6–24.

This leaves 240 participants (35.45%) who did not fit within the Alvanon sizing scheme, suggesting that they would likely experience difficulties in selecting a well-fitting garment. These findings highlight potential limitations in standardised sizing systems, emphasizing the need for more adaptable approaches to accommodate diverse body shapes.

### SVM model performance in predicting size via key measurements

The dataset was divided into training and test sets to develop the model and evaluate its performance. In this study, 70% of the data were allocated to the training set for training the SVM model, whereas 30% of the data were used as the test set to evaluate the model’s predictive accuracy.

#### Accuracy

The SVM model, trained to predict body size via key measurements (bust, waist, and hip), achieved an accuracy of 89.66%. This result demonstrates its strong ability to classify 677 participants accurately on the basis of their key body measurements and assign them to the appropriate size categories.

#### Confusion matrix

The confusion matrix (Table 4) summarises the model’s performance on the test set, illustrating how accurately it predicted each clothing size category.

**Table 4:** SVM confusion matrix for key measurements.

	Predicted_6	Predicted_8	Predicted_10	Predicted_12	Predicted_14	Predicted_16	Predicted_18	Predicted_20	Predicted_22	Predicted_24	Predicted_26	Predicted_28	Predicted_32	Predicted_34	Predicted_40
Actual_ 6	0	1	0	0	0	0	0	0	0	0	0	0	0	0	0
Actual_ 8	0	11	1	0	0	0	0	0	0	0	0	0	0	0	0
Actual_10	0	0	41	1	0	0	0	0	0	0	0	0	0	0	0
Actual_12	0	0	1	43	1	0	0	0	0	0	0	0	0	0	0
Actual_14	0	0	0	1	46	2	0	0	0	0	0	0	0	0	0
Actual_16	0	0	0	0	2	21	2	0	0	0	0	0	0	0	0
Actual_18	0	0	0	0	0	0	10	0	0	0	0	0	0	0	0
Actual_20	0	0	0	0	0	0	0	5	1	0	0	0	0	0	0
Actual_22	0	0	0	0	0	0	0	0	2	0	0	0	0	0	0
Actual_24	0	0	0	0	0	0	0	0	2	2	0	0	0	0	0
Actual_26	0	0	0	0	0	0	0	0	0	0	0	3	0	0	0
Actual_28	0	0	0	0	0	0	0	0	0	0	0	1	0	0	0
Actual_32	0	0	0	0	1	0	0	0	0	0	0	0	0	0	0
Actual_34	0	0	0	0	1	0	0	0	0	0	0	0	0	0	0
Actual_40	0	0	0	0	1	0	0	0	0	0	0	0	0	0	0

The confusion matrix was analysed to compare actual versus predicted classifications, providing insights into the effectiveness of the SVM model for multiclass classification tasks. The SVM model exhibited varying levels of accuracy across different size categories. Notably, common sizes (10, 12, and 14) achieved high accuracy rates, with 41, 43, and 46 correct predictions, respectively. However, classification challenges emerged in several areas: misclassifications were observed between adjacent sizes (e.g., size 8 was occasionally predicted as size 10), suggesting feature overlap at category boundaries; lower accuracy was recorded for extreme sizes (6, 32–40), primarily owing to limited representation in the training dataset; and a significant class imbalance was detected, particularly for sizes 26–40, affecting the model’s ability to predict these categories accurately.

These findings highlight the challenges associated with distinguishing closely related size categories in automated garment sizing systems, emphasizing the need for improved feature selection and balanced training datasets to increase predictive accuracy.

#### The territorial map and decision boundary

**Fig. 3:**
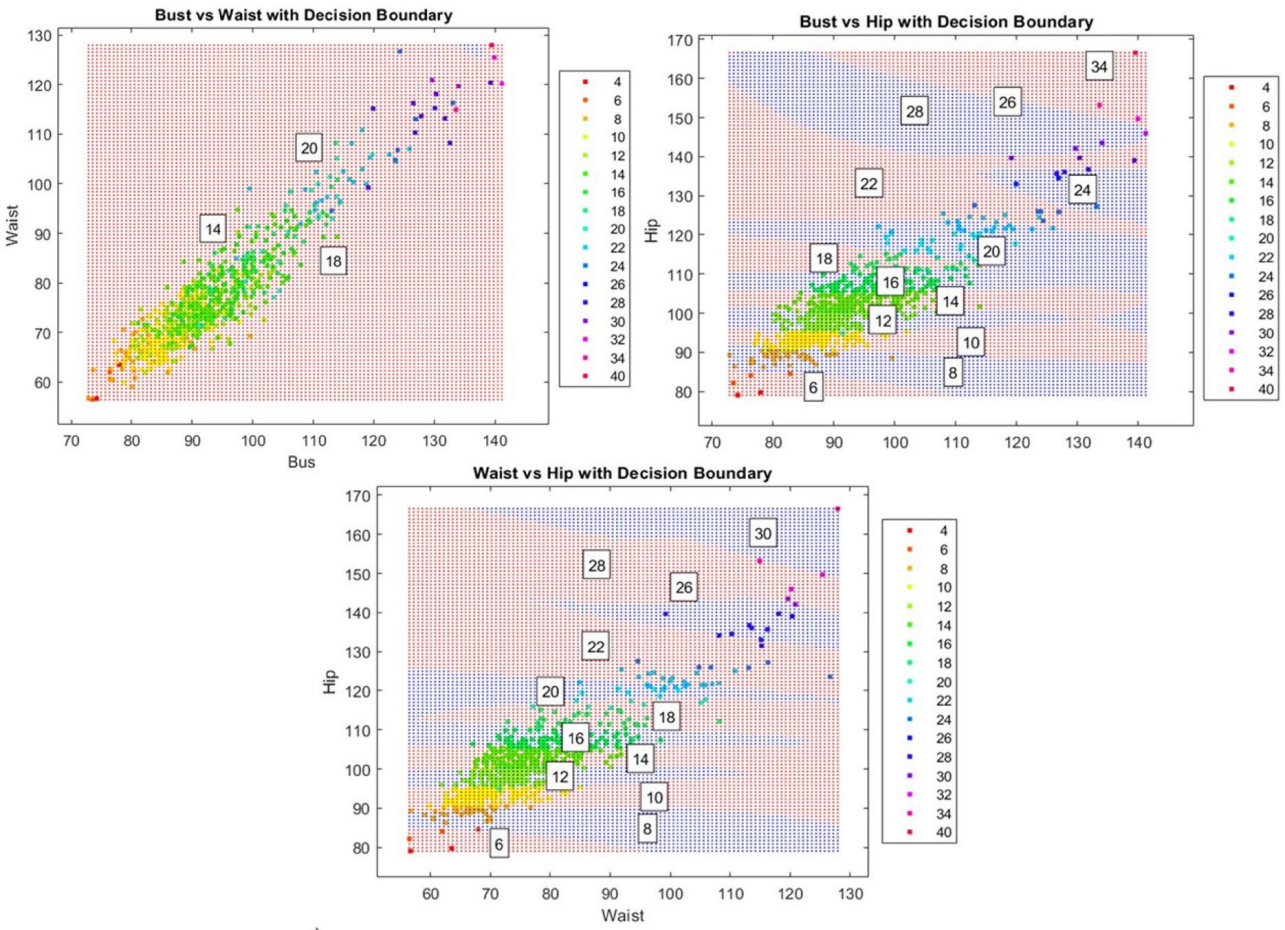
SVM decision boundaries for size classification based on different body measurements: bust vs. waist (top left), bust vs. hip (top right), and waist vs. hip (bottom), highlighting separation patterns across these measurement combinations.

The SVM classifier generates a territorial map in a 2D feature space, utilizing the RBF kernel transformation to establish nonlinear decision boundaries across multiple anthropometric projections (Bust-Waist, Bust-Hip, and Waist-Hip). Figure 3 illustrates these classification boundaries through color-coded regions (e.g., blue and red), highlighting the SVM’s hyperplane optimization for class separation. The cluster centroids (sizes 8–40) demonstrate distinct partitioning patterns, further emphasizing the model’s classification structure. Notably, classification complexity arises in the Bust-Waist projection, particularly within sizes ranging from 12 to 18, where dense clustering necessitates precise boundary determination. However, the model effectively manages measurement continuity across adjacent sizes (12–20), with Bust–Waist correlations emerging as primary determinants of classification boundaries.

### The SVM model size prediction using height and weight

The combination of height and weight identified by^28^ as the most reliable predictor of various body measurements was utilised in this study as an independent variable for predicting body size. This modelling experiment compared the results with key body measurements to assess its predictive effectiveness.

#### The accuracy

The SVM model, which was designed to predict body size via height and weight, achieved an accuracy of 51.72%. This result suggests a relatively weak classification performance, indicating a limited ability to accurately categorise the 677 participants into their predicted body sizes on the basis of only these two variables.

#### Confusion matrix

The confusion matrix (Table 5) illustrates the model’s performance on the test set, displaying the accuracy of the predictions for each clothing size category.

**Table 5:** SVM confusion matrix for height and weight.

	Predicted_8	Predicted_10	Predicted_12	Predicted_14	Predicted_16	Predicted_18	Predicted_20	Predicted_22	Predicted_24	Predicted_26	Predicted_28	Predicted_30	Predicted_32	Predicted_34
Actual_8	6	5	0	0	0	0	0	0	0	0	0	0	0	0
Actual_10	4	27	9	0	0	0	0	0	0	0	0	0	0	0
Actual_12	0	7	30	7	1	0	0	0	0	0	0	0	0	0
Actual_14	0	4	14	16	13	2	0	1	0	0	0	0	0	0
Actual_16	0	1	4	5	20	1	0	0	0	0	0	0	0	0
Actual_18	0	0	0	0	8	2	0	0	0	0	0	0	0	0
Actual_20	0	2	0	0	0	1	1	0	0	0	0	0	0	0
Actual_22	0	0	0	0	0	0	0	2	1	0	0	0	0	0
Actual_24	0	0	0	1	0	0	0	3	0	0	0	0	0	0
Actual_26	0	0	0	0	0	0	0	0	0	0	1	0	0	0
Actual_28	0	0	0	0	0	0	0	1	0	0	1	0	0	0
Actual_30	0	0	0	0	0	0	0	0	0	0	0	0	1	0
Actual_32	0	0	0	0	0	0	0	0	0	0	0	0	0	0
Actual_34	0	0	0	0	0	0	0	0	0	0	1	0	0	0

The SVM model generates a confusion matrix, where most correct predictions align along the diagonal. For example, size 10 is correctly predicted 27 times, whereas size 12 is correctly classified 30 times. However, misclassifications are observed, particularly for size 14, which is frequently misclassified as size 12 or 16. Similarly, size 16 is misclassified as size 14 (5 instances) and size 12 (4 instances). Additionally, sizes 32 and 34 received no correct predictions, suggesting that the model struggles with these categories because of overlapping features and insufficient training samples.

#### The territorial map

**Fig. 4:**
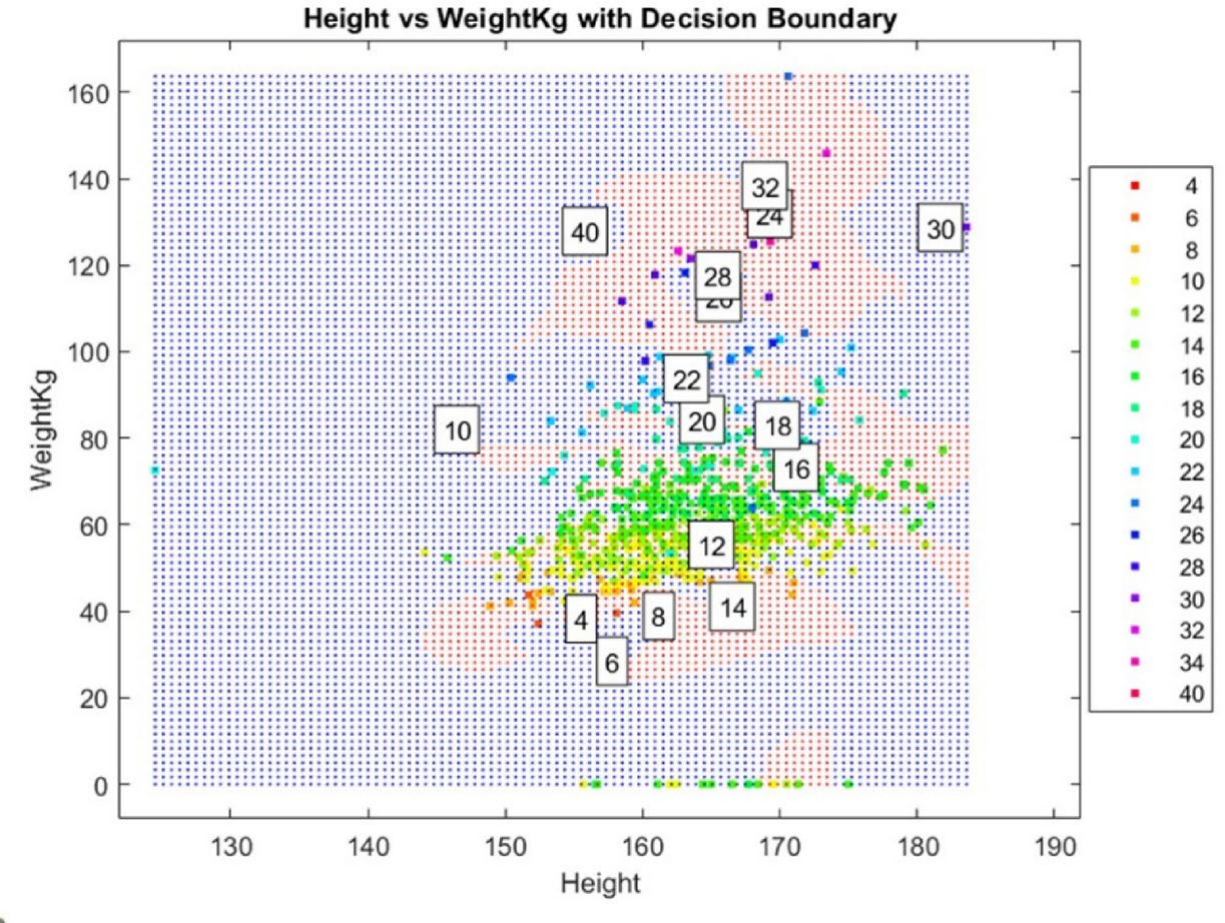
Decision boundaries based on height and weight.

Figure 4 illustrates the decision boundaries generated by the SVM model for body size prediction on the basis of height and weight. However, overlapping regions between different body size categories indicate classification challenges, suggesting that the model struggles to clearly differentiate adjacent sizes using only height and weight. This overlap likely results from the natural variability in body shape, which height and weight alone cannot be fully captured.

### SVM model for size prediction via multiple body measurements

Before the SVM algorithm was applied, principal component analysis (PCA) was performed to identify the most significant body features for representing body size and shape. This step aimed to develop an efficient machine learning model for body size prediction on the basis of data-driven anthropometric measurements.

#### Data suitability for factor analysis

The suitability of the dataset for factor analysis was assessed via two key statistical tests:

1. *Kaiser‒Meyer‒Olkin (KMO)* The KMO value was 0.904, which is considered excellent for PCA. As this value exceeds 0.6, it confirms that the sample is adequate and that factor analysis is appropriate for this dataset^60^.
2. *Bartlett’s test of sphericity* The test was statistically significant (p < 0.001), confirming that the correlation matrix is not an identity matrix. This result indicates strong relationships among the variables, supporting their suitability for factor analysis^61^.

These findings validate the use of factor analysis for feature extraction in anthropometric data, reinforcing its role in enhancing size prediction models.

#### Variance explained by principal components

Table 6 indicates that PCA identified three principal components that collectively explained 86.425% of the total variance in the dataset. This high cumulative variance suggests that these components capture the majority of the information contained in the original variables (body measurements), leading to a substantial reduction in dimensionality while preserving most of the dataset’s variability.

**Table 6:** PCA total variance.

Total variance explained									
Component	Initial eigenvalues			Extraction sums of squared loadings			Rotation sums of squared loadings		
	Total	% of Variance	Cumulative %	Total	% of Variance	Cumulative %	Total	% of Variance	Cumulative %
1	18.298	52.279	52.279	18.298	52.279	52.279	15.326	43.789	43.789
2	10.468	29.908	82.187	10.468	29.908	82.187	11.704	33.439	77.229
3	1.483	4.238	86.425	1.483	4.238	86.425	3.219	9.197	86.425

#### Scree plot

**Fig. 5:**
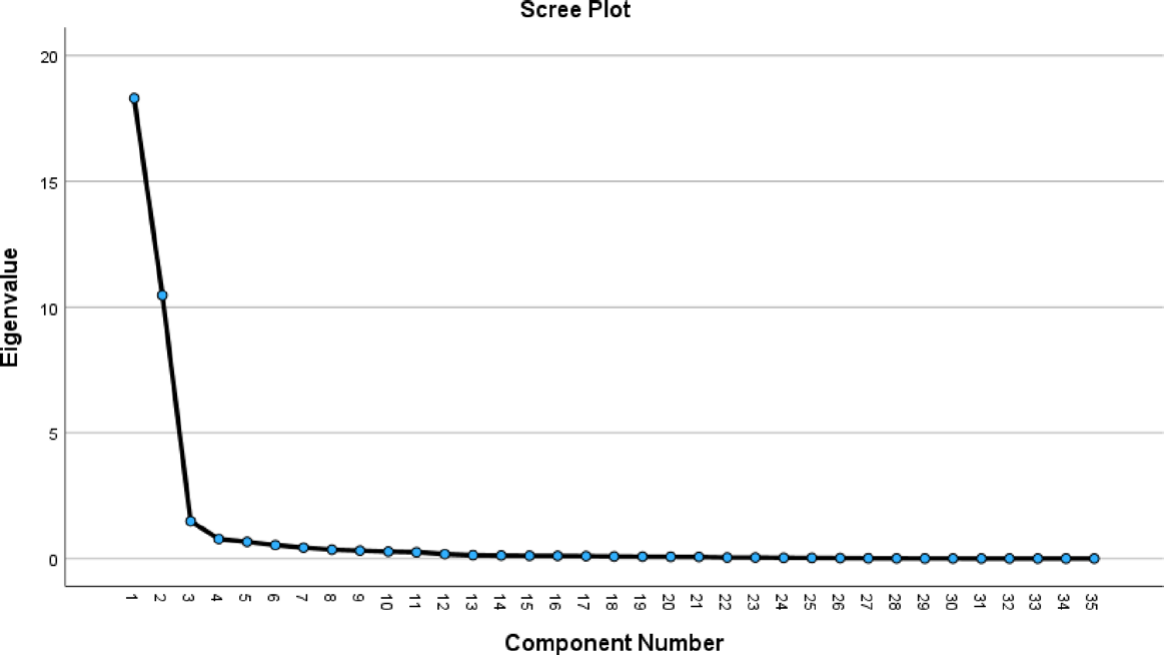
Scree plot.

The scree plot (Fig. 5) confirms the significance of the first three principal components, displaying a sharp elbow at the third component, followed by a gradual decline. This pattern supports the decision to use three principal components for analysis.

#### Rotated component matrix

The rotated component matrix (Table 7) provides insight into the underlying structure of the body measurements:

1. *Component 1*: This component is heavily influenced by circumference and arc measurements, such as waist, chest/bust, seat, hip, and shoulder measurements. It primarily represents key horizontal body measurements.
2. *Component 2* This component is associated with height-related measurements, including those of the shoulder, neck, crotch, and various vertical body heights. It clearly represents the vertical dimension or height factor of the body.
3. *Component 3* Although less prominent, this component appears to capture aspects of body width, particularly in the hip and thigh regions. It may represent a shape factor that differentiates body types independently of overall size.

**Table 7:** PCA rotated component matrix.

Rotated component matrix^a^			
	Component		
	1	2	3
Waist_ Circ	0.962		
Chest/Bust_Circ	0.946		
Waist_Circ_Bk_Arc	0.936		
Waist_Circ_Fr_Arc	0.930		
Shoulder_Circ	0.921		
Waist_SoB_2_Circ_Width	0.909		
ChestBust_Circ_Fr_Arc	0.886		
Seat_Circ	0.864		
Seat_Circ_Fr_Arc	0.855		
Snp_R_to_Bust_R	0.846		
Hip_CircTM_085c	0.839		
Hip_Fr_Arc_085f.	0.838		
Snp_L_to_Bust_L	0.836		
ChestBust_Circ_Bk_Arc	0.835		
Seat_Circ_Bk_Arc	0.790		
Thigh_Circ	0.782		
Seat_Circ_Width	0.772		
Hip_Bk_Arc	0.727		
Bust_Width	0.704		
Neck_Base_Circ	0.658		
Shoulder_Circ_Height		0.978	
Shoulder_Height		0.970	
Neck_Base_Side_Neck_Height_L		0.969	
Neck_Base_Side_Neck_Height_R		0.969	
Shoulder_Height		0.968	
Crotch_Height		0.956	
Thigh_Circ_Height		0.956	
ChestBust_Circ_Height		0.949	
Waist _Circ_Height		0.940	
Height cm		0.938	
Seat_Circ_Height		0.933	
Knee_Circ_Height		0.892	
Hip_Circ_Height_		0.752	
Thigh_MidLeg_Width			0.696
Hip_Circ_Width_			0.684

#### SVM model using PCA for extended measurements

##### The accuracy

After applying PCA for dimensionality reduction on 35 body dimensions, a new set of body features was generated, representing horizontal, vertical, and lower body measurements. The SVM model utilised these features to predict body size, achieving an accuracy of 68.97%. The model classified 677 participants on the basis of measurements influencing garment fit.

##### Confusion matrix

The PCA-SVM classification model (Table 8) demonstrated high accuracy within sizes 8–18, with peak performance observed in the 10–14 range. While some sizes showed strong classification performance (e.g., size 10: 85.3%, size 12: 73.6%, size 14: 80.5%), others experienced lower accuracy rates, particularly size 8 (43.75%), highlighting the challenges of consistent classification across all size categories. The overall model performance was evaluated by summing the correctly classified instances along the diagonal of the confusion matrix. With 140 correct predictions from a total of 203 test samples (by applying Eq. (3)), the PCA-SVM model achieved an overall accuracy of 68.97%.

**Table 8:** Confusion matrix for the PCA-SVM model.

	Predicted_ 6	Predicted_ 8	Predicted_10	Predicted_12	Predicted_14	Predicted_16	Predicted_18	Predicted_20	Predicted_22	Predicted_24	Predicted_26	Predicted_28	Predicted_30	Predicted_34
Actual_ 6	0	1	0	0	0	0	0	0	0	0	0	0	0	0
Actual_ 8	0	7	9	0	0	0	0	0	0	0	0	0	0	0
Actual_10	0	1	29	4	0	0	0	0	0	0	0	0	0	0
Actual_12	0	0	7	39	7	0	0	0	0	0	0	0	0	0
Actual_14	0	0	0	6	33	2	0	0	0	0	0	0	0	0
Actual_16	0	0	0	0	8	17	2	0	0	0	0	0	0	0
Actual_18	0	0	0	0	0	4	6	0	0	0	0	0	0	0
Actual_20	0	0	0	0	0	0	1	3	3	0	0	0	0	0
Actual_22	0	0	0	0	0	0	0	2	5	0	0	0	0	0
Actual_24	0	0	0	0	0	0	0	0	2	1	0	0	0	0
Actual_26	0	0	0	0	0	0	0	0	0	0	0	1	0	0
Actual_28	0	0	0	0	0	0	0	0	0	0	0	0	0	0
Actual_30	0	0	0	0	1	0	0	0	0	0	0	1	0	0
Actual_34	0	0	0	0	1	0	0	0	0	0	0	0	0	0

However, classification errors were noted along adjacent size boundaries, particularly within the 8–10–12–14 sequence. The model’s effectiveness was constrained by class imbalance and data sparsity, resulting in lower accuracy for extreme sizes (6 and 28–34) and larger categories (20 and above). These findings highlight limitations in feature representation and decision boundary optimization, particularly for underrepresented size groups.

##### The territorial map

**Fig. 6:**
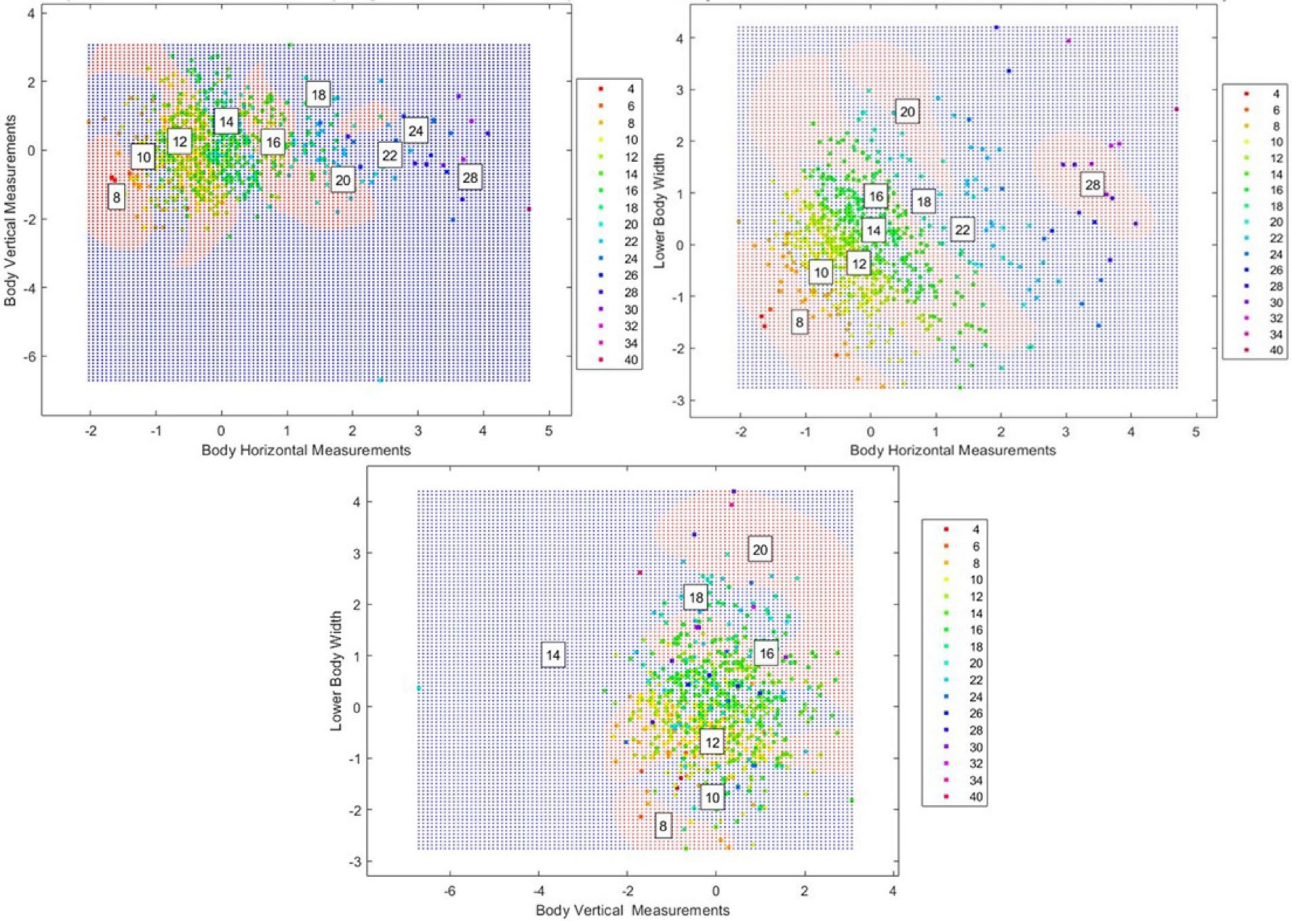
PCA-SVM decision boundaries for size classification on the basis of 35 body measurements. (Top left) Body horizontal vs. vertical measurements; (Top right) Body horizontal vs. lower body width; (Bottom) Body vertical vs. lower body width.

Figure 6 (top left) illustrates a strong positive correlation between horizontal and vertical body measurements, where smaller sizes (4–10) cluster in the lower-left quadrant, whereas larger sizes (28–40) appear in the upper-right quadrant. As the number of horizontal measurements increases, the number of vertical measurements also tends to rise, although not consistently across all sizes. Figure 6 (top right) reveals a clearer relationship between body circumference and lower body width, showing significant overlap among midrange sizes (12–20). These findings suggest that lower body width plays a crucial role in size variability within these categories. Figure 6 (bottom) highlights complex interactions between body height and lower body width, demonstrating substantial overlap among size categories. This underscores the challenges of defining distinct classification boundaries in automated garment sizing systems.

## Discussion

### Models performance analysis and body size classification variability

The findings reveal that while key measurement analysis shows a transition from smaller to larger sizes, substantial variability exists among individuals within each size category. This underscores the complexity of designing sizing systems that accommodate diverse body shapes. The misalignment occurs because variability in body shape cannot be fully captured by a single average form. Unlike retailer-standard forms, which rely on simplified average data with limited direct measurements, real body shapes exhibit a greater degree of diversity. The primary objective of this study was to assess the effectiveness of SVM models in predicting body size via a set of anthropometric measurements.

**Fig. 7:**
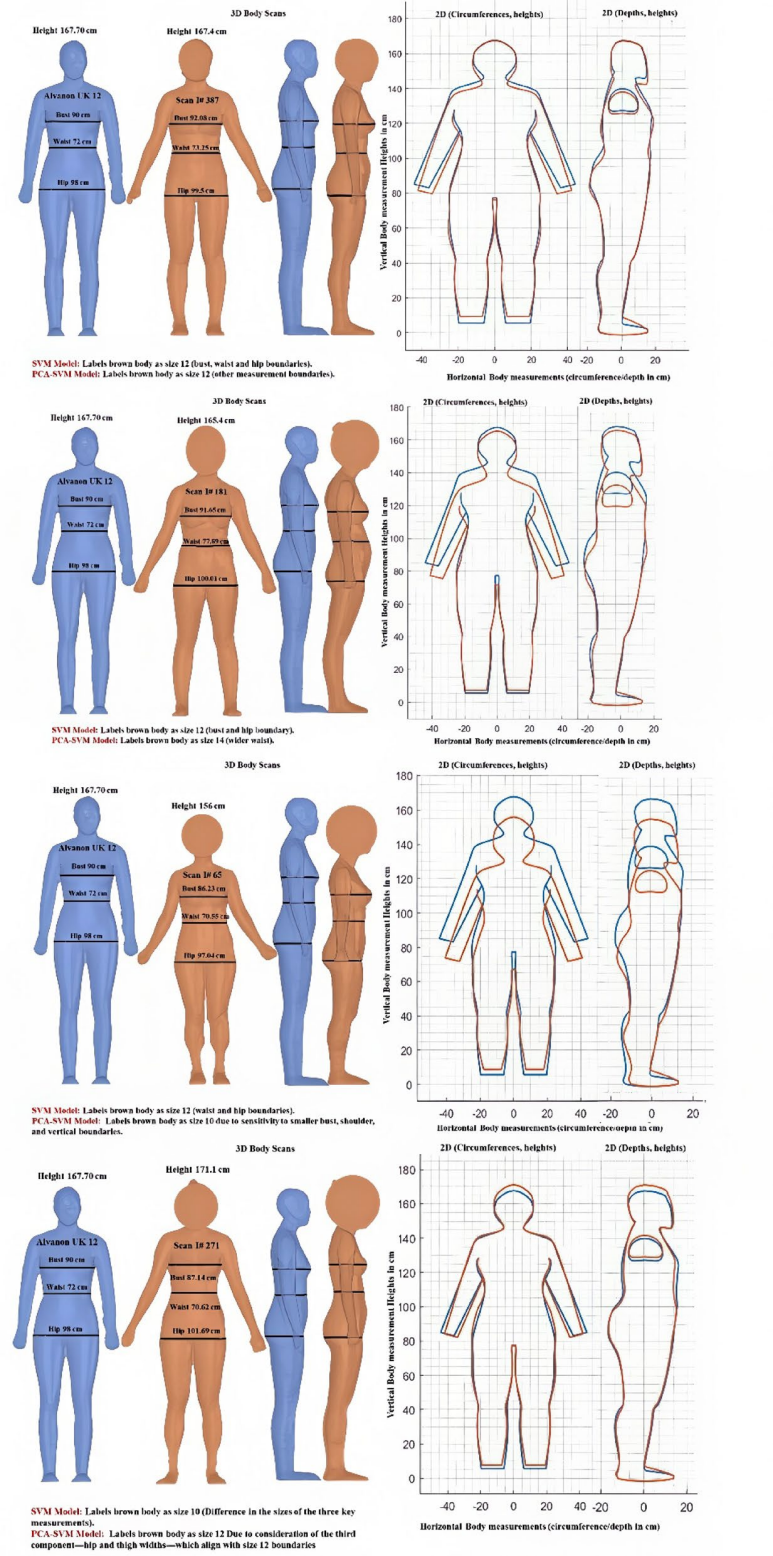
Visual comparison of SVM and PCA-SVM size prediction.

A visual comparison of body outlines across four cases revealed distinct behavioral differences between the SVM and PCA-SVM implementations. The analysis was conducted in relation to the standard Alvanon UK size 12 model (height: 167.70 cm, bust: 90 cm, waist: 72 cm, hip: 98 cm), as illustrated in Fig. 7. Case 1 demonstrates high accuracy in size 12 prediction by both models, validating the SVM’s effectiveness for standard body measurements. Case 2 reveals the sensitivity of PCA-SVM to proportional variations, with a prediction size of 14 due to an increased waist circumference, whereas the SVM maintains a size of 12 classifications on the basis of primary measurements. Case 3 illustrates the dimensional sensitivity of PCA-SVM, which predicts size 10 due to reductions in bust, shoulder width, and vertical measurements, despite standard hip measurements. Case 4 highlights the models’ divergent approaches—SVM predicts size 14 on the basis of increased hip measurements, whereas PCA-SVM assigns size 12, reflecting its comprehensive analysis of body proportion relationships. This discrepancy underscores the third principal component’s influence on lower body dimensions. These findings demonstrate the enhanced ability of PCA-SVM to capture subtle variations in body shape beyond traditional measurement parameters.

### Interpretation of model performance

The SVM model achieved an accuracy of 89.66%. While short of perfect, this figure represents a good result for a baseline model operating on complex, real-world anthropometric data. Given the inherent biological variability and the continuous nature of body measurements, some degree of overlap between size categories is unavoidable.

An important finding in this study is the performance difference between the standard SVM and the PCA-SVM model. Although the standard SVM achieves higher classification accuracy by using original anthropometric measurements, the PCA-SVM model offers distinct advantages in understanding body shape variation. PCA reduces dimensionality by identifying directions of greatest variance, effectively capturing underlying body proportion patterns that may not be evident from individual measurements alone. This allows the PCA-SVM model to recognise complex interrelationships between dimensions, such as proportional differences between bust, waist, and hip, which are critical in garment fit but may be overlooked in models focused solely on absolute values. As demonstrated in the case studies (Fig. 7), PCA-SVM is particularly effective in identifying subtle shape variations that influence size classification, even when key individual measurements fall within standard thresholds. This indicates that PCA-SVM provides a more holistic approach to body modelling and may offer superior performance in applications where overall shape and proportion are central to garment design and fit.

### Limitations and generalisability

It is important to acknowledge several limitations that may affect the generalisability of this study’s findings. First, the dataset of 677 participants, while diverse, is modest in size relative to the broader consumer population. Consequently, the research results may not fully capture the complete spectrum of body shape variations. Second, the dataset exhibits a significant class imbalance, with a concentration of participants in the mid-range sizes (e.g., 8–22) (as showen in Table 3) and very few representatives in the extreme sizes (e.g., 6, 24, and above). As observed in our confusion matrices, this data sparsity directly impacts the models’ predictive accuracy for these underrepresented categories. While the findings regarding the superiority of the standard SVM are strong for the core sizes, its performance on extreme sizes is less reliable. Third, the dataset exhibits age distribution skewness (median: 24 years, mean: 29 years, range: 18–77 years), with predominant representation of younger participants. However, this demographic characteristic does not affect the validity of our primary research objective. The study evaluates SVM effectiveness in predicting clothing sizes based on current anthropometric measurements a classification task that relies on measured body dimensions rather than age as a predictor variable. Since body measurements themselves inherently capture the physical characteristics relevant to garment fit regardless of the age at which they were acquired, the age distribution does not compromise model performance assessment or the comparative analysis between SVM and PCA-SVM approaches. The models classify individuals into size categories based solely on their measurements making age-related morphological differences already reflected in the measurement data itself rather than requiring age as a separate model input. Therefore, while this study provides a baseline, the development of generalised, production-ready sizing models will necessitate future work with substantially larger and more demographically balanced datasets.

## Conclusion

This study evaluated the performance of Support Vector Machine (SVM) algorithms and a hybrid PCA-SVM approach in predicting clothing sizes based on anthropometric data extracted from 3D body scans. Analysing measurements from 677 participants, the research identified significant challenges in size prediction due to diverse body morphologies and the inadequacy of traditional sizing systems in representing nuanced body dimensions. The findings indicate that 35.45% of participants did not align with any specific sizing category, emphasizing the need for machine learning (ML) and artificial intelligence (AI) approaches to enhance size prediction accuracy. The SVM model, which relies on key measurements such as bust, waist, and hip, achieved an accuracy of 89.66%, whereas the PCA-SVM model, which incorporates a broader range of measurements, attained an accuracy of 68.97%. This discrepancy underscores the complexities associated with integrating additional variables into classification models. However, this study has several limitations. Although the dataset is diverse, it may not fully capture all ethnic body shape variations, potentially introducing biases in size prediction. Additionally, class imbalance, particularly in extreme size categories, may limit the model’s generalizability. While machine learning techniques offer a promising approach to size prediction, further validation in real-world fitting scenarios is required to ensure their practical applicability.

To address these gaps, future work should prioritise expanding datasets to include underrepresented body sizes, mitigating bias, and enhancing model robustness. A critical path for future work is to benchmark these SVM-based models against the current state-of-the-art methods discussed in the literature review, such as deep learning architectures. This direct comparison would provide a clear understanding of the trade-offs between this study model’s interpretability and the potential predictive gains from more complex, data-intensive approaches. Additionally, feature engineering and further hyperparameter optimization such as k-fold cross-validation and SVM hyperparameter tuning could continue to refine the model’s capabilities for intricate body shape classification tasks.

## Data Availability

The datasets generated and/or analysed during the current study are not publicly available due to ethical restrictions and participant privacy protections, as the data are from a private body scan data set collected for research purposes and managed by the Apparel Design Engineering group at the University of Manchester under specific ethical approval conditions that prohibit public sharing in their current form. Appropriately processed data may be available from the corresponding author on reasonable request. Inquiries may be directed to Dr Simeon Gill (simeon.gill@manchester.ac.uk).

## References

[1] Du, E. S. J., Liu, C. & Wayne, D. H. Automated Fashion Size Normalization; Preprint at http://arxiv.org/abs/1908.09980 (2019).

[2] Gill, S. & Sanderson, R. Redefining the drawn body through investigations of proportional techniques. *5th Inter-disciplinary Glob. Conf. Fash.* (2013).

[3] Gill, S. A review of research and innovation in garment sizing, prototyping and fitting. *Text. Prog.* **47**(1), 1–85. 10.1080/00405167.2015.1023512 (2015).

[4] Kuo, C. C., Wang, M. J. & Lu, J. M. Developing sizing systems using 3D scanning head anthropometric data. *Meas. J. Int. Meas. Confed.* **152**, 107264. 10.1016/j.measurement.2019.107264 (2020).

[5] Chi, C., Zeng, X., Bruniaux, P. & Tartare, G. An intelligent recommendation system for personalised parametric garment patterns by integrating designer’s knowledge and 3D body measurements. *Ergonomics*10.1080/00140139.2024.2332772 (2024). 38544443 10.1080/00140139.2024.2332772

[6] David Kreyenhagen, C., Aleshin, T. I., Bouchard, J. E., Wise, A. M. I. & Zalegowski, R. K. Using supervised learning to classify clothing brand styles. In *2014 IEEE Systems and Information Engineering Design Symposium, SIEDS 2014* vol. 00, no. c 239–243 (2014). 10.1109/SIEDS.2014.6829909.

[7] Ashmawi, S., Alharbi, M., Almaghrabi, A. & Alhothali, A. FITME: Body measurement estimations using machine learning method. *Procedia Comput. Sci.* **163**, 209–217. 10.1016/j.procs.2019.12.102 (2019).

[8] Van Vugt, J. Human body prediction of size and shape A hormonal framework. *ACM Int. Conf. Proc. Ser.*10.1145/3386164.3387262 (2019).

[9] Zhang, J., Luximon, Y., Shah, P., Zhou, K. & Li, P. Customize my helmet: A novel algorithmic approach based on 3D head prediction. *CAD Comput. Aided Des.* **150**, 103271. 10.1016/j.cad.2022.103271 (2022).

[10] Eshel, Y., Levi, O., Roitman, H. & Nus, A. *PreSizE: Predicting Size in E-Commerce using Transformers* vol. 1, no. 1 (Associationfor Computing Machinery, 2021). 10.1145/3404835.3462844.

[11] Liu, K., Zhu, C., Tao, X., Bruniaux, P. & Zeng, X. Parametric design of garment pattern based on body dimensions. *Int. J. Ind. Ergon.* **72**(June), 212–221. 10.1016/j.ergon.2019.05.012 (2019).

[12] Meyer, P., Birregah, B., Beauseroy, P., Grall, E. & Lauxerrois, A. Missing body measurements prediction in fashion industry: a comparative approach. *Fash. Text.*10.1186/s40691-023-00357-5 (2023).

[13] Cortes, C. & Vapnik, V. Support-vector networks. vol. 297, pp. 273–297 (1995).

[14] Vapnik, V. C. C. Support-vector networks. vol. Mach Learn, no. 20, pp. 273–297 (1995). 10.1007/BF00994018

[15] Zhang, S. Research on young females’ body classification based on SVM. *Acad. J. Sci. Technol.* **4**(3), 73–76. 10.54097/ajst.v4i3.4790 (2022).

[16] Demšar, U., Harris, P., Brunsdon, C., Fotheringham, A. S. & McLoone, S. Principal component analysis on spatial data: an overview. *Ann. Assoc. Am. Geogr.* **103**(1), 106–128. 10.1080/00045608.2012.689236 (2013).

[17] Zakaria, N., Taib, J. S. M. N., Tan, Y. Y. & Wah, Y. B. Using data mining technique to explore anthropometric data towards the development of sizing system. In *Proceedings—International Symposium on Information Technology 2008, ITSim* vol. 2 (2008). 10.1109/ITSIM.2008.4631721.

[18] Varte, R. L. et al. Data mining anthropometric parameters for the design and sizing of female full body protector. *Def. Life Sci. J.* **6**(4), 275–283. 10.14429/DLSJ.6.16602 (2021).

[19] Baytar, F. & Forstenhausler, L. N. Dressed to the form: An examination of dress form asymmetry and its relation to garment fit. no. 2013, pp. 3–5 (2020). 10.31274/itaa.12140.

[20] Chan, A. C. K. *The Development of Apparel Sizing Systems from Anthropometric Data* (Woodhead Publishing Limited, 2014). 10.1533/9780857096890.2.167.

[21] Apeagyei, P. R. Significance of body image among UK female fashion consumers: The cult of size zero, the skinny trend. *Int. J. Fash. Des. Technol. Educ.* **1**(1), 3–11. 10.1080/17543260701867697 (2008).

[22] Lee, Y. et al. The definition and generation of body measurements (ISO 8559 Series of Standards). In *Proceedings of the 20th Congress of the International Ergonomics Association (IEA 2018)* 405–422 (2019). 10.1007/978-3-319-96089-0_20 PMC10658266 37987021

[23] Staples, M. L. & Delury, D. B. A system for the Sizing of Women’s Garments. *Text. Res. J.* **19**(6), 346–354. 10.1177/004051754901900605 (1949).

[24] Ashdown, S. P. *Creation of ready-made clothing: the development and future of sizing systems* (Woodhead Publishing Limited, 2014). 10.1533/9781782422150.1.17.

[25] Gill, S., Januszkiewicz, M. & Ahmed, M. *7. Digital fashion technology: A review of online fit and sizing* (2022). 10.1016/B978-0-12-823969-8.00008-3.

[26] Beazley, A. Size and fit: fomulation of body measurement tables and sizing systems. II. *J. Fash. Mark. Manag.* **2**(3), 260–284. 10.1108/eb022534 (1998).

[27] Chun-Yoon, J. & Jasper, C. R. Key dimensions of women’s ready-to-wear apparel: Developing a consumer size-labeling system. *Cloth. Text. Res. J.* **14**(1), 89–95. 10.1177/0887302X9601400111 (1996).

[28] Winks, J. *Clothing Sizing, International Standardization* (The Textile Institute, 1997).

[29] Lovato, C., Milanese, C., Giachetti, A. & Zancanaro, C. 3D digital anthropometry using the BodySCAN. no. October, pp. 259–263 (2010). 10.15221/10.259.

[30] Daanen, H. A. M. & Psikuta, A. *3D Body Scanning*, vol. 1, no. 2017 (Elsevier Ltd, 2018). 10.1016/B978-0-08-101211-6.00010-0.

[31] Sohn, J. M., Lee, S. & Kim, D. E. An exploratory study of fit and size issues with mass customized men’s jackets using 3D body scan and virtual try-on technology. *Text. Res. J.* **90**(17–18), 1906–1930. 10.1177/0040517520904927 (2020).

[32] Wang, Z., Zhong, Y. Q., Chen, K. J., Ruan, J. Y. & Zhu, J. C. 3D human body data acquisition and fit evaluation of clothing. *Adv. Mater. Res.* **989–994**, 4161–4164. 10.4028/www.scientific.net/AMR.989-994.4161 (2014).

[33] Verikas, A., Gelzinis, A. & Bacauskiene, M. Mining data with random forests: A survey and results of new tests. *Pattern Recognit.* **44**(2), 330–349. 10.1016/j.patcog.2010.08.011 (2011).

[34] Ranbaduge, T., Vatsalan, D. & Christen, P. *Clustering-based scalable indexing for multi-party privacy-preserving record linkage*, vol. 9078 (2015). 10.1007/978-3-319-18032-8_43.

[35] Darst, B. F., Malecki, K. C. & Engelman, C. D. Using recursive feature elimination in random forest to account for correlated variables in high dimensional data. *BMC Genet.* **19**(Suppl 1), 1–6. 10.1186/s12863-018-0633-8 (2018). 30255764 10.1186/s12863-018-0633-8 PMC6157185

[36] Zhao, J., He, X. & Kemao, Q. Automatic body measurement by neural networks. In *ACM International Conference Proceeding Series* 9–14 (2019). 10.1145/3378891.3378897.

[37] Liu, K. et al. An evaluation of garment fit to improve customer body fit of fashion design clothing. *Int. J. Adv. Manuf. Technol.* **120**(3–4), 2685–2699. 10.1007/s00170-022-08965-z (2022).

[38] Dik, N. Y., Tsang, P. W. K., Chan, A. P., Lo, C. K. Y. & Chu, W. C. A novel approach in predicting virtual garment fitting sizes with psychographic characteristics and 3D body measurements using artificial neural network and visualizing fitted bodies using generative adversarial network. *Heliyon* **9**(7), e17916. 10.1016/j.heliyon.2023.e17916 (2023). 37483761 10.1016/j.heliyon.2023.e17916 PMC10362334

[39] Liu, K., Wang, J., Kamalha, E., Li, V. & Zeng, X. Construction of a prediction model for body dimensions used in garment pattern making based on anthropometric data learning. *J. Text. Inst.* **108**(12), 2107–2114. 10.1080/00405000.2017.1315794 (2017).

[40] Wang, Z., Wang, J., Xing, Y., Yang, Y. & Liu, K. Estimating human body dimensions using RBF artificial neural networks technology and its application in activewear pattern making. *Appl. Sci.*10.3390/app9061140 (2019).32944385

[41] Portillo Juan, N., Matutano, C. & Negro Valdecantos, V. Uncertainties in the application of artificial neural networks in ocean engineering. *Ocean Eng.* **284**(March), 115193. 10.1016/j.oceaneng.2023.115193 (2023).

[42] Fan, F. L., Xiong, J., Li, M. & Wang, G. On interpretability of artificial neural networks: A survey. *IEEE Trans. Radiat. Plasma Med. Sci.* **5**(6), 741–760. 10.1109/TRPMS.2021.3066428 (2021). 35573928 10.1109/trpms.2021.3066428 PMC9105427

[43] Rathore, B. Artificial intelligence in sustainable fashion marketing: Transforming the supply chain landscape. *Eduzone Int. Peer Rev. Acad. Multidiscip. J.* **08**(02), 25–38. 10.56614/eiprmj.v8i2y19.363 (2019).

[44] Salawu, E. O. et al. Using artificial neural network to predict body weights of rabbits. *Open J. Anim. Sci.* **04**(04), 182–186. 10.4236/ojas.2014.44023 (2014).

[45] Costa, E., Silva, E., Rocha, H., Maia, A. & Vieira, T. Study on machine learning algorithms to automatically identifying body type for clothing model recommendation. In *World Conference on Information Systems and Technologies* 74–84 (Springer, 2018).

[46] Wang, Z. et al. Construction of garment pattern design knowledge base using sensory analysis, ontology and support vector regression modeling. *Int. J. Comput. Intell. Syst.* **14**(1), 1687–1699. 10.2991/IJCIS.D.210608.002 (2021).

[47] Pal, M. & Foody, G. M. Evaluation of SVM, RVM and SMLR for accurate image classification with limited ground data. *IEEE J. Sel. Top. Appl. Earth Obs. Remote Sens.* **5**(5), 1344–1355. 10.1109/JSTARS.2012.2215310 (2012).

[48] Hsu, C. H., Lin, H. F. & Wang, M. J. Developing female size charts for facilitating garment production by using data mining. *J. Chin. Inst. Ind. Eng.* **24**(3), 245–251. 10.1080/10170660709509039 (2007).

[49] Fu, B., Zheng, R., Chen, Q. & Zhang, Y. An improved clothing size recommendation approach based on subdivision of female body types. *Ergonomics*10.1080/00140139.2022.2069867 (2022). 35471127 10.1080/00140139.2022.2069867

[50] Vapnik, V. N. *Statistical Learning Theory* (Wiley, 1998).

[51] Tian, Y., Qi, Z., Ju, X., Shi, Y. & Liu, X. Nonparallel support vector machines for pattern classification. *IEEE Trans. Cybern.* **44**(7), 1067–1079. 10.1109/TCYB.2013.2279167 (2014). 24013833 10.1109/TCYB.2013.2279167

[52] Hearst, M. A., Dumais, S. T., Osuna, E., Platt, J. & Scholkopf, B. Support vector machines. *IEEE Intell. Syst. their Appl.* **13**, 18–28. 10.1109/5254.708428 (1998).

[53] Cristianini, N. & Shawe-Taylor, J. *An Introduction to Support Vector Machines and Other Kernel-based Learning Methods* (Cambridge University Press, 2000). https://books.google.co.uk/books?id=_PXJn_cxv0AC

[54] Kecman, V. Support vector machines—An introduction 1 basics of learning from data. *Theory Appl.* **177**, 1–47. 10.1007/10984697_1 (2005).

[55] Parker, C. J., Gill, S., Harwood, A., Hayes, S. G. & Ahmed, M. A method for increasing 3D body scanning’s precision: Gryphon and consecutive scanning. *Ergonomics* **65**(1), 39–59. 10.1080/00140139.2021.1931473 (2022). 34006206 10.1080/00140139.2021.1931473

[56] Shlens, J. Shlens2006_PCATutorial. *Measurement* 1–13 (2005). papers3://publication/uuid/4D1DBE59-7625-4528-BAB6-E076486F0C77.

[57] Patle, A. & Chouhan, D. S. SVM kernel functions for classification. *2013 Int. Conf. Adv. Technol. Eng. ICATE 2013* 1–9 (2013). 10.1109/ICAdTE.2013.6524743.

[58] Lopez, C. P. *Big Data and Deep Learning. Examples with Matlab*. CESAR PEREZ (2020).

[59] Escalera, S., Pujol, O. & Radeva, P. Error-correcting ouput codes library. *J. Mach. Learn. Res.* **11**, 661–664 (2010).

[60] Kaiser, H. F. An index of factorial simplicity. *Psychometrika* **39**, 31–36 (1974).

[61] Knapp, T. R. & Swoyer, V. H. Some Empirical Results concerning the Power of Bartlett’s Test of the Significance of a Correlation Matrix Published by : American Educational Research Association Stable. https://www.jstor.org/stable/1161720 REFERENCES Linked references are availa,” vol. 4, no. 1, pp. 13–17, 1967.

[62] Alvanon. Alvanon Standard UK WOMEN. vol. 44, no. 0, p. 4 (2015). www.alvanon.com.

